# Comparative analysis of AQP7 expression and cryotolerance in X- and Y-chromosome bearing bovine sperm

**DOI:** 10.3389/fcell.2025.1582961

**Published:** 2025-05-16

**Authors:** Jieru Wang, Ruchun Li, Fei Huang, Peng Niu, Yanling Zheng, Dongfang Yuan, Yan Du, Congcong Li, Mingcai Yang, Jiajia Suo, Qinghua Gao

**Affiliations:** ^1^ Department of Veterinary Medical Science, Shandong Vocational Animal Science and Veterinary College, Weifang, Shandong, China; ^2^ College of Life Sciences and Technology, Tarim University, Alar, Xinjiang, China; ^3^ College of Animal Sciences and Technology, Tarim University, Alar, Xinjiang, China

**Keywords:** AQP7, AQP3, cryotolerance of sperm, X-sperm, Y-sperm

## Abstract

**Introduction:**

The livestock industry has witnessed a notable increase in the proportion of male calves born following artificial insemination with cryopreserved semen, hinting at a possible association between cryotolerance and inherent structural or functional disparities between X- and Y-chromosome bearing sperm. To delve into this phenomenon, we conducted a comprehensive proteomic analysis on bovine sex-sorted semen.

**Methods:**

Sperm samples were meticulously categorized based on stringent quality parameters. Concurrent sorting of X- and Y-sperm from identical ejaculates was performed, followed by protein extraction for subsequent analysis. Quantitative isobaric tags for relative and absolute quantification (iTRAQ) were employed to identify differentially expressed proteins.

**Results:**

iTRAQ pinpointed 20 proteins with differential expression between X- and Y-spermatozoa, including 12 upregulated and 8 downregulated proteins in Y-sperm. Aquaporin 7 (AQP7), significantly upregulated in Y-sperm, was identified as a key candidate. Western blot analysis corroborated elevated AQP7 expression in Y-sperm compared to X-sperm, while Aquaporin 3 (AQP3) showed no significant difference. Immunofluorescence revealed AQP7 localization to the post-acrosomal region, midpiece, and principal piece of Y-sperm with higher fluorescence intensity, whereas AQP3 distribution was comparable between groups.

**Discussion:**

These findings imply a potential role for AQP7 in augmenting Y-sperm cryotolerance, providing molecular insights into sex-specific cryopreservation effects on fertility outcomes. Future research should elucidate AQP7’s functional mechanisms and explore implications for livestock breeding technologies.

## 1 Introduction

In livestock production, improving reproductive efficiency and controlling offspring sex are critical objectives for both researchers and producers. Artificial insemination (AI) has become an indispensable tool in modern breeding, significantly enhancing reproductive success and enabling sex selection (Moore and Hasler, 2017). However, cryopreservation of semen remains a challenge due to the adverse effects of freezing and thawing, which can compromise sperm viability and fertilizing potential (Fujii et al., 2018). A slight imbalance in the male-to-female offspring ratio, typically around 51%–52% males to 48%–49% females (Mendes et al., 2022), is commonly observed in AI with frozen semen. This deviation suggests that Y sperm may exhibit greater cryotolerance, which could influence the efficiency of sexed semen production.

Sperm cryotolerance directly impacts fertilization success and the accuracy of sex control, making it a critical area of focus for optimizing sexed semen production. Aquaporins (AQPs), particularly AQP3 and AQP7, have emerged as key regulators of sperm membrane function, playing essential roles in water and glycerol transport during cryopreservation (Prieto-Martínez et al., 2017). AQP7, in particular, has been implicated in the enhanced cryotolerance of Y sperm, as evidenced by studies in dairy cattle and goats, where its expression is significantly higher in Y sperm compared to X sperm.

Despite these findings, comprehensive studies on the expression differences of AQP7 across breeds and its molecular mechanisms in sperm cryotolerance remain lacking. This study aims to investigate the expression of AQP7 in sexed semen from different cattle breeds and explore its role in enhancing sperm cryotolerance. Specifically, we will (Moore and Hasler, 2017): assess the post-thaw quality of sexed spermatozoa using both conventional and advanced parameters (Fujii et al., 2018); identify and analyze the proteomic differences between X- and Y-bearing sperm; and (Mendes et al., 2022) investigate the expression levels and localization patterns of AQP3 and AQP7 in sexed spermatozoa. Through these investigations, we aim to provide valuable insights into the molecular basis of sperm cryotolerance and contribute to the development of improved cryopreservation protocols, ultimately enhancing the efficiency of sexed semen production and the precision of sex control in livestock breeding.

## 2 Materials and methods

This study was conducted from August 2022 to December 2024. The experimental procedures were under the Regulations of the Administration of Affairs Concerning Experimental Animals (Ministry of Science and Technology, Beijing, China, 2004), and were approved by the Animal Ethics Committee of Tarim University, and the approval number was TARU-2022–004.

### 2.1 Experimental design

This study employed to compare cryotolerance and proteomic differences between X- and Y-bearing sperm from Angus, Simmental, and Wagyu bulls. Ejaculates meeting strict quality criteria were divided into two fractions for concurrent X/Y sorting via fluorescence-activated cell sorting (FACS), ensuring intra-ejaculate control. Sorted sperm (≥90% purity) and non-sorted controls were cryopreserved identically. Post-thaw quality (motility, viability, acrosome integrity, mitochondrial activity) was assessed using CASA and fluorescent staining. Comparative proteomics (LC-MS/MS) analyzed X vs. Y sperm proteins, with key targets validated by Western blot and immunofluorescence.

### 2.2 Animals and grouping

The experimental animals used in this study were sourced from Shandong OX Livestock Breeding Co., Ltd (Shandong, China), including Angus, Simmental, and Wagyu bulls. Breed abbreviations were as follows: AG for Angus, XM for Simmental, and HE for Wagyu. All animals were in healthy condition, with similar physiological characteristics, including age and weight, to ensure the accuracy and reliability of the results. To control for the effect of bull, semen samples were collected from multiple bulls, and the ejaculates were split into X and Y sperm fractions simultaneously using a split ejaculate design. The animals were subjected to strict quarantine and adaptive feeding protocols to minimize any potential environmental factors that could affect the study outcomes. Bulls were provided a nutritionally balanced diet based on their physiological needs, with *ad libitum* access to water and appropriate veterinary care.

The X- and Y-chromosome bearing sperm were sorted concurrently from the same ejaculate, and grouped based on sperm quality parameters.

### 2.3 Semen collection and cryopreservation

#### 2.3.1 Semen collection and sex-sorting procedure

Semen was collected twice a week using an artificial vagina. In the experimental design, three bulls were selected from each breed (Angus, Simmental, and Wagyu) to ensure biological diversity. For each bull, ejaculate samples were collected with a minimum of five replicates (n ≥ 5 per bull) to account for individual variability and meet statistical power requirements. Ejaculates were immediately assessed for volume, sperm concentration, and progressive motility using a computer-assisted sperm analysis (CASA) system (AndroVision, Minitube, Germany). Only ejaculates with motility ≥80%, concentration ≥1.0 × 10^9^ sperm/mL, and <10% morphological abnormalities were included. For each ejaculate, the semen was split into two equal parts, and each part was sorted separately into X and Y sperm fractions using fluorescence-activated cell sorting.

For sex-sorting, sperm was incubated with Hoechst 33,342 dye (5 μg/mL) at 34°C for 45 min, with simultaneous sorting of X- and Y-chromosome bearing sperm to stain DNA. The stained sperm were then sorted by fluorescence-activated cell sorting (FACS) using a high-speed flow cytometer (SX MoFlo, Becton Dickinson, United States). Sperm X/Y sorting was performed under optimized parameters: UV laser excitation at 355 nm for Hoechst 33,342-stained DNA detection, forward/side scatter voltages of 150–300 V, nozzle diameter 70–100 μm, sheath pressure 20–25 psi, and sorting rate ≤5,000 events/sec, achieving ≥90% purity through strict gating based on the 3.8% DNA content difference between X- and Y-chromosome-bearing sperm. The sorted sperm fractions were collected, with X- and Y-sperm fractions coming from the same ejaculate, diluted with a Tris-based extender (0.2 M Tris, 0.07 M citric acid, 0.06 M glucose, 20% (v/v) Specific Pathogen Free egg yolk, 1% (v/v) penicillin-streptomycin solution, and 6.4% (v/v) glycerol), achieving a final sperm concentration of 20 × 10^6^ sperm/mL. After cooling to 4°C for 2 h, sperm were loaded into 0.25-mL straws. This process was performed separately for each split ejaculate sample to ensure that the X and Y sperm fractions from the same ejaculate were processed under identical conditions.

#### 2.3.2 Cryopreservation and thawing

The cryopreservation process involved sealing and labeling the loaded straws, followed by a controlled-rate freezing protocol in a programmable biological freezer. This protocol included an initial cooling phase from −4°C to −80°C over 112 s (40 °C/min), a 30-s hold at −80°C for thermal equilibrium, and a secondary cooling phase from −80°C to −150°C over 70 s (60°C/min). Finally, the straws were transferred to liquid nitrogen vapor phase (−196°C) for long-term storage.

For thawing, straws were incubated in a 37°C water bath for 30 s and used immediately for further analysis.

### 2.4 Evaluation of post-thaw sperm quality

#### 2.4.1 Sperm motility and kinematic parameters

Post-thaw motility and progressive motility were assessed using the CASA system (AndroVision, Minitube, Germany). Kinematic parameters, including curvilinear velocity (VCL), straight-line velocity (VSL), average path velocity (VAP), amplitude of lateral head displacement (ALH), beat cross frequency (BCF), working velocity (WOB), linearity (LIN), and straightness (STR), were analyzed to evaluate sperm movement patterns and efficiency. The sperm quality assessment using the AndroVision system was performed with the following settings: a CASA-specific sperm counting chamber was utilized, and the temperature was maintained at 38°C using a heated stage. For each sample, approximately 1,000 sperm were analyzed per field, and a total of 5 fields were evaluated to ensure comprehensive and representative data collection.

#### 2.4.2 Viability

Sperm viability was assessed by adding Hoechst33342/PI (15,407/0,009, Minitube, Germany) to the semen, incubating at 38°C for 15 min, and examining under a microscope. Red staining indicates membrane damage, while blue staining indicates intact membrane integrity. A minimum of three replicates were recorded, with each replicate analyzing at least 1,000 sperm. The CoolLED device of AndroVision system controller was set with the following light intensity configurations for analysis: 1UV at 100%, 2B at 100%, and 3 GR at 0%/OFF.

#### 2.4.3 Acrosome integrity

Acrosome integrity was evaluated by adding Hoechst33342/FITC-PNA (15,407/0,011, Minitube) to the semen, incubating at 38°C for 20 min, and observing the fluorescence under a microscope. Green fluorescence in the acrosome region indicates acrosome damage, while only blue fluorescence throughout the sperm indicates intact acrosome integrity. A minimum of three replicates were recorded, with each replicate analyzing at least 1,000 sperm. The CoolLED device of AndroVision system controller was set with the following light intensity configurations for analysis: 1UV at 69%, 2B at 77%, and 3 GR at 0%/OFF.

#### 2.4.4 Mitochondrial activity

Mitochondrial activity was assessed by adding Hoechst33342/Rhodamine123 (15,407/0,012, Minitube) to the semen, incubating at 38°C for 20 min. The sample was centrifuged at 800 *g* for 4 min at room temperature (approximately 25°C) in the dark. After centrifugation, the supernatant was discarded, and 50 μL of extender was added to the pellet. Green fluorescence in the middle segment of the sperm indicates strong mitochondrial activity, while its absence suggests weak or no mitochondrial activity. A minimum of three replicates were recorded, with each replicate analyzing at least 1,000 sperm. The CoolLED device of AndroVision system controller was set with the following light intensity configurations for analysis: 1UV at 100%, 2B at 100%, and 3 GR at 0%/OFF.

### 2.5 Protein extraction

#### 2.5.1 Sperm protein extraction

For protein extraction, SDS-free protein RIPA buffer was added to the sperm samples, along with a cocktail protease inhibitor (cOmplete™, Roche) at a final concentration of 1×. After incubating on ice for 5 min, Dithiothreitol (DTT, 10 mM final concentration) was added, and the samples were disrupted using an ultrasonic processor (amplitude: 40%, pulse: 10 s on/10 s off). After lysis, the samples were centrifuged at 25,000g for 15 min at 4°C to collect the supernatant. The supernatant was treated with DTT (10 mM final concentration) and incubated at 37°C for 30 min, followed by the addition of Iodoacetamide (IAM, 55 mM final concentration) and incubation in the dark for 45 min. The samples were then precipitated with acetone (5× volume) at −20°C for 2 h, followed by centrifugation and air-drying. The protein pellet was dissolved in SDS-free protein lysis buffer, and the samples were centrifuged again to collect the protein solution.

#### 2.5.2 Protein quantification

Protein concentration was determined using a gradient concentration protein standard. A 5 μL aliquot of various protein standards was added to a 96-well plate. A 5 μL aliquot of the sample was added to the sample wells. If the sample volume was less than 5 μL, it was supplemented with standard dilution solution to reach 5 μL. After adding 250 μL of G250 dye solution to each well, absorbance was measured at A595 using a microplate reader. The protein concentration was calculated based on the standard curve and sample volume (Bradford Protein Assay Kit, Beyotime, China).

### 2.6 LC-MS/MS analysis

#### 2.6.1 Protein digestion

Protein (100 μg) was digested in a 10 kDa ultrafiltration tube. The samples were centrifuged, and the supernatant was removed. Trypsin was added at a 1:20 ratio, and the samples were incubated at 37°C for 4 h. The digestion solution was collected, and the process was repeated for additional washing and concentration.

#### 2.6.2 Peptide labeling

IBT reagent (2 mg) was dissolved in 80 μL isopropanol and mixed for 1 min. The digested peptides were dissolved in 0.2 M TEAB to achieve a concentration of 4 μg/μL, then mixed with 80 μL IBT reagent. The mixture was incubated at room temperature for 2 h for full labeling.

#### 2.6.3 Peptide separation

Peptides were separated using an Agilent LC-20AD liquid chromatography system with a Gemini C18 column (5 μm, 20 cm × 180 μm). A gradient elution method was used with solvents A (5% ACN, pH 9.8) and B (95% ACN, pH 9.8).

#### 2.6.4 Mass spectrometry detection

Peptides were ionized by nanoESI and analyzed using a Q-Exactive HF X tandem mass spectrometer (Thermo Fisher Scientific) in Data Dependent Acquisition (DDA) mode. The ion source voltage was set to 1.9 kV, and the resolution was 60,000 for the first scan. Fragmentation was performed with HCD mode, and spectra were analyzed in an Orbitrap analyzer with a resolution of 30,000.

#### 2.6.5 Bioinformatics analysis

Bioinformatics analysis was performed using the SwissProt/UniProt *Bos taurus* protein database and NCBI databases, including *Bos angus × Brahman*, *Bos javanicus*, *Bos mutus*, *Bos hereford*, *Bos wagyu*, and *Bos zebu*.

### 2.7 Western blot analysis

Proteins were separated by SDS-PAGE (10% gels, 150 V for 35 min) and transferred to PVDF membranes (260 mA for 90 min). After blocking with 5% non-fat milk, the membranes were incubated with primary antibodies against AQP3 (1:3,000, bs-1253R; Bioss, China) and AQP7 (1:3,000, bs-2506R; Bioss) overnight at 4°C. HRP-conjugated secondary antibodies (1:5,000, S0001; Affinity, China) were applied, and bands were visualized using enhanced chemiluminescence (ECL) reagents (Bio-Rad, United States). Band intensity was analyzed using ImageJ software (v1.53k). Briefly, 8-bit grayscale conversion and background subtraction (rolling ball radius = 50 pixels) were applied to reduce noise. Bands of interest were identified by automatic thresholding (Default algorithm) with manual adjustment to exclude non-specific signals. Densitometry was performed by measuring the Integrated Density and Area of target bands and GAPDH (Suo et al., 2024).

### 2.8 Immunofluorescence

For localization studies, Sperm samples were smeared onto microscope slides, air-dried, and fixed with 4% paraformaldehyde for 30 min at room temperature, permeabilized with 0.1% Triton X-100, and blocked with 5% BSA. Primary antibodies against AQP3 and AQP7 were applied (1:120, Tris-Buffered Saline with Tween 20 (TBST)) overnight at 4°C, followed by Alexa Fluor 594-conjugated secondary antibodies (1:250 TBST, S0006, Affinity). Nuclei were counterstained with DAPI, and slides were imaged using a Nikon Ti2-U fluorescence microscope. Fluorescence intensity was quantified using ImageJ (v1.53k). Immunofluorescence images were converted to 8-bit grayscale, and background noise was subtracted (rolling ball radius = 50 pixels). Automatic thresholding (Default algorithm) defined regions of interest, with manual adjustments to exclude non-specific signals. For each sample, 5 random fields (each field contains no less than 200 sperm) were analyzed, and fluorescence intensity was calculated as the mean gray value (Integrated Density/Area).

### 2.9 Statistical analysis

Data were analyzed using GraphPad Prism 9 (GraphPad Software, United States). Normality was assessed using the Shapiro-Wilk test. Parametric data were analyzed using one-way ANOVA followed by Tukey’s *post hoc* test, while non-parametric data were analyzed using the Kruskal–Wallis test. Results are presented as mean ± standard error of measurement (SEM), with differences considered significant at p < 0.05.

## 3 Results

### 3.1 XY sperm quality parameters

Six *B. taurus* samples (Samples AG-4600-X, XM-9321-X, and HE-5104-X corresponded to X sperm, whereas AG-4600-Y, XM-9321-Y, and HE-5104-Y represented Y sperm) underwent three repeated, and the sperm quality parameters of X and Y sperm selected for the experiment are presented in Table 1.

**TABLE 1:** Sperm quality parameters of X and Y sperm.

Parameter	Unit	X sperm	Y sperm
Motility	%	52.84 ± 1.86^a^	63.22 ± 2.43^b^
Progressive Motility	%	38.46 ± 0.33^a^	44.59 ± 1.56^b^
VCL	μm/s	47.37 ± 4.39	52.35 ± 2.66
VSL	μm/s	19.11 ± 0.90	21.55 ± 0.81
VAP	μm/s	24.32 ± 0.56	25.94 ± 0.62
ALH	μm	0.5520 ± 0.0041	0.6450 ± 0.0935
BCF	Hz	7.34 ± 0.98	9.95 ± 1.07
WOB	%	52.80 ± 3.89	48.50 ± 2.60
LIN	%	41.20 ± 2.01	38.50 ± 3.57
STR	%	78.40 ± 2.06	79.00 ± 3.42
Viability	%	54.92 ± 2.65	51.95 ± 1.11
Mitochondrial Activity	%	54.37 ± 2.22	52.69 ± 0.86
Acrosome Integrity	%	58.50 ± 2.44	58.32 ± 0.58

Data are presented as mean ± standard error of measurement (SEM). Superscript letters indicate significant differences between groups (p < 0.05).

### 3.2 Protein concentration and quality control of XY sperm

Protein concentrations were measured using the Bradford method, and the standard curve generated was described by the regression equation y = 2.2524x + 0.015, with an *R* ^2^ value of 0.9917, indicating a strong linear correlation between absorbance and protein concentration (Supplementary Figure S1). Based on the standard curve, the protein concentrations and total protein amounts for the sperm samples are shown in Table 2.

**TABLE 2:** Protein concentration and total protein amounts of XY sperm.

Sample	Protein concentration (μg/μL)	Volume (μL)	Total protein (μg)
AG 4600 X	7.91	280	2,214.20
XM 9321 X	14.19	280	3,973.80
HE 5104 X	12.19	280	3,412.90
AG 4600 Y	14.66	280	4,105.26
XM 9321 Y	12.81	280	3,586.52
HE 5104 Y	10.72	280	3,000.46

SDS-PAGE analysis was conducted to assess the integrity and reproducibility of the sperm protein samples. Supplementary Figure S2 shows the SDS-PAGE results, where the protein markers indicate a molecular weight distribution from 12 kDa to 120 kDa. The gel revealed clear and reproducible bands for the X and Y sperm samples, indicating good protein integrity.

### 3.3 Mass spectrometry quality control

Mass spectrometry results showed the highest number of peptide ions with scores between 10 and 30 (Supplementary Figure S3A), indicating a high degree of match between peptides and protein sequences, providing a robust dataset for further protein quantification. The coefficient of variation (CV) for repeat experiments was 0.15 (Supplementary Figure S3B), confirming the high reproducibility of the method. Protein molecular weight distributions showed a broad range (Supplementary Figure S3C), suggesting the identification of diverse proteins, which expands the range of protein detection. Most peptide lengths were between 7–20 amino acids (Supplementary Figure S3D), which are stable and detectable, supporting subsequent structural and functional analyses.

### 3.4 XY sperm differential protein analysis

In the IBT quantitative analysis, 6 *Bos taurus* samples (AG-4600-X, XM-9321-X, HE-5104-X, AG-4600-Y, XM-9321-Y, HE-5104-Y) underwent three repeated experiments, generating a total of 700,442 secondary spectra. Using a 1% FDR filter in the Uniprot database, 274 peptides and 128 proteins were identified; in the NCBI database, 270 peptides and 131 proteins were identified.

#### 3.4.1 Protein identification and functional annotation

Gene Ontology (GO) annotations were performed using both the Uniprot and NCBI databases, revealing that all identified sperm proteins play important roles in fertilization processes, including catalytic activity, binding activity, transport activity, and involvement in cell proliferation, differentiation, and fertilization (Supplementary Figure S4). Differences in GO annotations between the two databases provided a more comprehensive view of the sperm proteins' functions.

#### 3.4.2 KOG annotation

KOG annotation from both Uniprot and NCBI databases revealed that all identified sperm proteins were primarily involved in cellular processes and signal transduction, metabolism and energy production, and information storage and processing. These categories highlight the essential roles of sperm proteins in cellular signaling, metabolic pathways, and gene expression regulation, which are crucial for sperm development, motility, and fertilization (Supplementary Figure S5).

#### 3.4.3 Pathway annotation

Pathway annotations in the KEGG database identified five major categories for all identified sperm proteins: metabolism, organic systems, human diseases (restricted to animals), environmental information processing, and cellular processes. Most sperm proteins were enriched in these pathways, indicating their involvement in critical reproductive processes, including energy supply, cellular metabolism, and signaling pathways. Additionally, some proteins were annotated in genetic information processing pathways, highlighting their potential roles in gene regulation and DNA replication (Supplementary Figure S6).

### 3.5 XY sperm differential protein analysis

iTRAQ protein quantification was used to analyze the differential protein expression between X and Y sperm, and Using X sperm as the control group. The analysis revealed 20 differential proteins, with 12 proteins upregulated in Y sperm and 8 proteins downregulated in Y sperm compared to X sperm. The upregulated proteins in Y sperm included Keratin 77 (KRT77), Peptide YY 2 (PYY2), KRT18, AQP7, KRT7, olfactory receptor, family 12, subfamily D (OR12D), Heat Shock Protein Beta 9 (HSPB9), Parkinson disease protein 7 (PARK7), Retinol-binding protein 4 (RET4), Acrosin, Recombinant Low Density Lipoprotein Receptor Related Protein 2 (LRP2), and Proteinase Inhibitor (PTI). The downregulated proteins included NEH4, Cyclin-dependent Kinase 3 (CDK3), Collagen, type I, alpha 1 (COL1A1), Caltrin precursor, Keratin-associated Protein (KAP2), ADP/ATP translocase4 (ADT4), Outer Dense Fiber of sperm tails 1 (ODFP1), and A-kinase anchoring protein (AKAP4). These results are summarized in Table 3.

**TABLE 3:** Differential proteins in X and Y sperm (Using X sperm as the control group).

Database sequence	Protein name	Differential expression
tr|G3MYU2|G3MYU2_BOVIN	KRT77	Upregulated
sp|P06833|PYY2_BOVIN	PYY2	Upregulated
tr|A1XEA5|A1XEA5_BOVIN	KRT18	Upregulated
NP_001069846.1	AQP7	Upregulated
NP_001039876.1	KRT7	Upregulated
tr|E1BLI6|E1BLI6_BOVIN	OR12D	Upregulated
sp|Q2TBQ6|HSPB9_BOVIN	HSPB9	Upregulated
sp|Q5E946|PARK7_BOVIN	PARK7	Upregulated
sp|P18902|RET4_BOVIN	RET4	Upregulated
tr|P79343|P79343_BOVIN	Acrosin	Upregulated
XP_070219633.1	LRP2	Upregulated
XP_027414228.1	PTI	Upregulated
tr|A0A3S5ZP41|A0A3S5ZP41_BOVIN	NEH4	Downregulated
XP_059744348.1	CDK3	Downregulated
XP_059740412.1	COL1A1	Downregulated
NP_776381.1	Caltrin precursor	Downregulated
sp|P00515|KAP2_BOVIN	KAP2	Downregulated
sp|Q2YDD9|ADT4_BOVIN	ADT4	Downregulated
XP_005899435.1	ODFP1	Downregulated
XP_027390338.1	AKAP4	Downregulated

#### 3.5.1 XY sperm differential protein clustering and volcano plots

**FIGURE 1:**
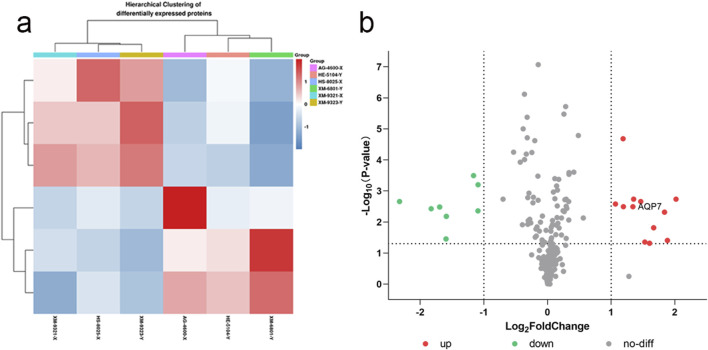
XY sperm differential protein clustering **(a)** and volcano plot **(b) (a)** Clustering analysis of differential proteins; **(b)** Volcano plot showing significant differential proteins based on fold change and p-value.

The differential proteins were further analyzed using clustering analysis and volcano plot visualizations, as shown in Figure 1.

#### 3.5.2 GO functional classification of differential proteins in XY sperm

From the clustering analysis and volcano plot visualizations, we observed that the differential proteins between X and Y sperm were significantly enriched in the cellular component category of Gene Ontology (GO) classification. Notably, these differential proteins exhibited significant upregulation in the cell membrane component (Supplementary Figure S7). This finding deepens our understanding of sperm gender differences at the molecular level and suggests that the cell membrane is a key site for differential protein expression. Given these findings, we selected **AQP7** (Aquaporin 7) as a focal point for further investigation. AQP7 plays a critical role in the cell membrane and was upregulated in Y sperm compared to X sperm. We hypothesize that AQP7 may be essential in sperm development, maturation, and function, and its differential expression could be linked to the sex determination mechanism in sperm. Therefore, we plan to focus on AQP7 for detailed functional analysis and mechanistic exploration, to uncover its specific role in XY sperm differentiation and its potential biological significance. This research will enhance our understanding of the molecular mechanisms underlying sperm gender differences and could offer new insights for the treatment and prevention of related reproductive disorders.

### 3.6 Western blot analysis of AQP3 and AQP7 in XY sperm

**FIGURE 2:**
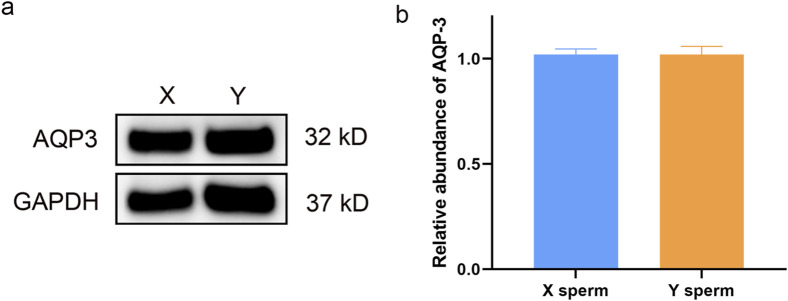
Western blot analysis of AQP3 in X and Y sperm **(a)** Western blot results; **(b)** Relative expression levels of AQP3 in different groups.

**FIGURE 3:**
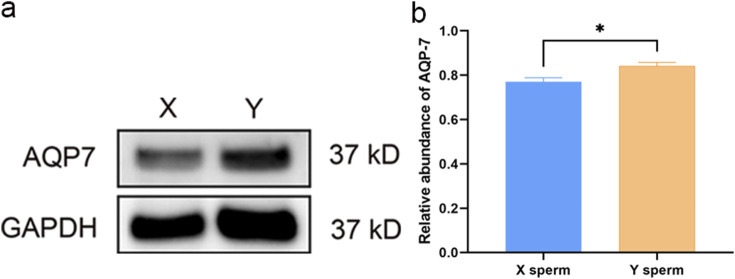
Western blot analysis of AQP7 in X and Y sperm **(a)** Western blot results; **(b)** Relative expression levels of AQP7 in different groups.

Western blot analysis revealed no significant differences in AQP3 expression between X and Y sperm. However, AQP7 expression was significantly higher in Y sperm compared to X sperm (Figures 2, 3).

### 3.7 Immunofluorescence localization of AQP3 and AQP7 in XY sperm

**FIGURE 4:**
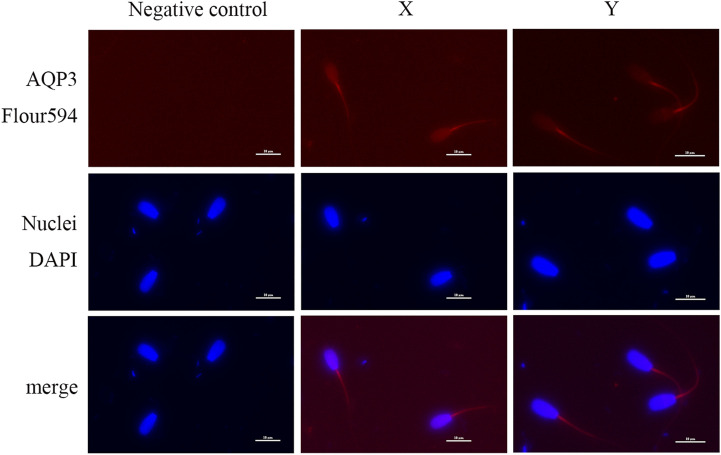
Subcellular localization of AQP3 Sperm nuclei are stained with blue fluorescence, while AQP3 is visualized with red fluorescence (scale bar = 10 μm).

**FIGURE 5:**
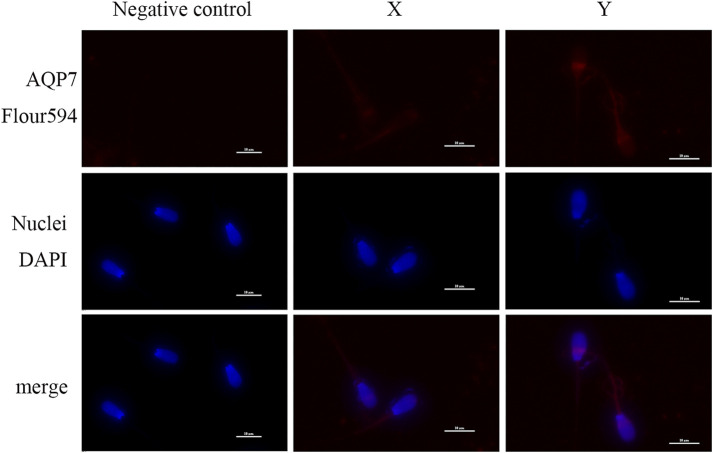
Subcellular localization of AQP7 Sperm nuclei are stained with blue fluorescence, while AQP7 is visualized with red fluorescence (scale bar = 10 μm).

Immunofluorescence analysis showed that AQP3 was primarily localized in the tail midpiece and principal piece of both X and Y sperm, with no significant differences in localization (Figure 4). In contrast, AQP7 exhibited enhanced fluorescence in the acrosomal region and the tail midpiece of Y sperm compared to X sperm (Figure 5).

## 4 Discussion

In livestock breeding, the control of offspring gender and the enhancement of reproductive efficiency are key areas of focus. Genetically, the mating of a bull with a cow theoretically results in an equal distribution of male and female offspring. However, observations in practical production often reveal deviations from this expected ratio, with the birth of more female calves in some natural breeding conditions, particularly noted in Irish farming traditions (Berry and Cromie, 2007). Although this observation aligns with certain cultural practices and agricultural preferences, it has not yet been fully substantiated by scientific evidence. However, with the rapid advancement of modern breeding techniques, especially artificial insemination (AI), a noticeable trend has emerged where AI significantly increases the ratio of male calves (Mendes et al., 2022; Arega and Chalchissa, 2019) This phenomenon has prompted further exploration into sperm characteristics, particularly the differences between X- and Y-bearing sperm.

One of the most significant factors influencing the outcome of sexed semen production is sperm cryotolerance, especially since sperm in AI typically undergoes cryopreservation. It has been hypothesized that Y sperm may exhibit superior cryotolerance compared to X sperm, thus offering a competitive advantage in fertilization, which in turn could influence the gender ratio of offspring. Studies focusing on Holstein cattle populations show a male-to-female ratio of approximately 52:48, which closely aligns with the general estimates for this breed (Berry and Cromie, 2007). In contrast, studies on beef cattle show varying gender ratios, with reports indicating a tendency for male calves to be slightly more prevalent than female calves, particularly with the use of AI, as observed in breeds such as the Charolais (Crews, 2006) and Angus (Berger et al., 1992). This suggests that the method of insemination, along with other environmental and biological factors, may influence the sex ratio.

Cryopreservation of sperm can cause cellular stress, leading to decreased viability and fertilization potential. Among various factors that impact sperm quality during freezing, the difference in cryotolerance between Y and X sperm has been a point of intense investigation. Berry and Cromie (2007)) reported a 1.24%–1.66% increase in the birth ratio of male calves with the use of frozen semen compared to fresh semen. This suggests that Y sperm may possess superior resistance to freezing stress, which could explain the higher success rate of male fertilization following cryopreservation. Optimizing the freezing process, particularly for Y sperm, could potentially improve sperm viability and enhance the accuracy of gender control.

Sexed semen production using flow cytometry allows for the sorting of X- and Y-bearing sperm. However, the process, which involves the use of fluorescent dyes, pressure, laser light, and sorting biases, can potentially affect sperm quality, leading to a reduction in vital sperm parameters such as motility and membrane integrity. Despite the theoretical precision of flow cytometry, the physical and chemical stresses associated with the sorting procedure can cause irreversible damage to the sperm, particularly affecting membrane proteins and mitochondrial functions Mostek et al., 2020). In our study, we observed that despite differences in vitality and motility, the advanced sperm parameters and overall sperm quality did not differ significantly between X and Y sperm, suggesting that the impact of the sorting process on sperm motility and functionality may be similar for both sperm types.

In our differential protein analysis, we identified 12 upregulated proteins in Y sperm, including KRT77, PYY2, KRT18, AQP7, KRT7, OR12D, HSPB9, PARK7, RET4, Acrosin, LRP2, and PTI. These proteins are involved in structural integrity, antioxidant activity, and sperm motility, which could explain the superior cryotolerance observed in Y sperm. For example, proteins like KRT77, KRT18, and KRT7 are part of the keratin family, known for their role in stabilizing cellular membranes. Their higher expression in Y sperm may help protect the sperm membrane from mechanical stress during the sorting and freezing processes, maintaining membrane stability and enhancing survival during thawing (Mostek et al., 2020; Lorente et al., 2021). HSPB9, a heat shock protein, is another important protein that was upregulated in Y sperm. Heat shock proteins are molecular chaperones that assist in protein folding and cellular repair under stress, and their increased expression may contribute to the enhanced resilience of Y sperm during the freezing and thawing processes (Gacem et al., 2023; Saadi et al., 2013). PARK7, an antioxidant and mitochondrial regulatory protein, may play a key role in protecting mitochondrial function during cryopreservation, thereby stabilizing energy metabolism and supporting sperm vitality (Zhang et al., 2023). Additionally, PYY2 (also known as Caltrin) is involved in calcium homeostasis and protects sperm from oxidative stress-induced membrane damage during freezing (San Agustin and Lardy, 1990). OR12D, an olfactory receptor protein, is significantly upregulated in Y sperm. This protein likely plays a role in signal transduction and intercellular communication, which could enhance the chemotaxis and fertilizing ability of Y sperm. Additionally, OR12D’s high expression may help reduce membrane lipid peroxidation induced by low temperatures during cryopreservation by participating in membrane remodeling and signaling (Ramirez-Diaz et al., 2023). Another upregulated protein in Y sperm is RET4, a retinol-binding protein involved in vitamin A metabolism and antioxidant processes. The high expression of RET4 could provide enhanced antioxidant protection for Y sperm, thus reducing the damage caused by reactive oxygen species (ROS) during freezing and thawing, and maintaining the integrity of both the sperm membrane and mitochondria (Kodzik et al., 2023). Acrosin, a critical protease in the acrosome, is also upregulated in Y sperm. This protein plays a vital role in the acrosome reaction and fertilization (Kasimanickam and Kasimanickam, 2024). Its increased expression could improve acrosome integrity and membrane stability, thereby enhancing the cryotolerance of Y sperm (Pinart et al., 2015). Furthermore, LRP2, a lipid metabolism-associated protein, is upregulated in Y sperm. This protein is involved in lipid transport and membrane repair, which is crucial during the freezing process, as the sperm membrane is a primary target for cryodamage. The increased expression of LRP2 may help stabilize the sperm membrane by facilitating lipid repair and enhancing overall sperm survival during cryopreservation (Argov et al., 2007). Similarly, PTI, a proteinase inhibitor, is upregulated in Y sperm. TPI plays a role in inhibiting the activity of trypsin and other serine proteases, which can otherwise damage sperm membranes during freezing. By inhibiting excessive protease activity, TPI protects membrane proteins from degradation, thus enhancing sperm cryotolerance (Cesari et al., 2004).

In contrast, our analysis also identified eight proteins that were downregulated in Y sperm, including NHE4, CDK3, COL1A1, Caltrin precursor, KAP2, ADT4, ODFP1, and AKAP4. NHE4, involved in pH regulation and ion balance within the cell, showed higher expression in X sperm. The higher expression of NHE4 in X sperm may contribute to its ability to better manage the pH changes during cryopreservation, thus reducing the damage caused by cellular acidification (Zhang et al., 2017). Similarly, CDK3, which plays a role in cell cycle regulation and DNA repair, is highly expressed in X sperm. This could be linked to DNA protection mechanisms during the freezing process (Pranomphon et al., 2024). COL1A1, an important extracellular matrix protein, was also more highly expressed in X sperm. It is involved in cell-matrix interactions and helps stabilize the sperm membrane, enhancing its structural integrity and potentially improving post-thaw survival rates (Elango et al., 2023). The Caltrin precursor protein, which regulates calcium ion flow, showed higher expression in X sperm, indicating its role in maintaining calcium balance during cryopreservation, thus protecting sperm function (Coronel et al., 1995). PRKAR2A, which regulates ATP generation for sperm motility, was expressed at higher levels in X sperm. This protein is critical for sustaining sperm forward motility and mitigating oxidative stress, both of which are crucial for sperm survival and fertilizing ability during cryopreservation (Zhang et al., 2022). ADT4, which is involved in energy metabolism and ATP synthesis in mitochondria, was also more highly expressed in X sperm, suggesting that X sperm may have a higher metabolic efficiency, aiding its recovery after cryopreservation (Ashrafzadeh et al., 2024). ODF1, a key component of the sperm tail’s outer dense fibers, plays a crucial role in maintaining the mechanical strength and elasticity of the tail, essential for sperm motility. The higher expression of ODF1 in X sperm may help preserve sperm structure under cryogenic stress, facilitating its motility and functional integrity post-thaw (Hinsch et al., 2004). Lastly, AKAP4, a major structural protein in the sperm tail, is associated with sperm motility and flagellar dynamics. The higher expression of AKAP4 in X sperm could contribute to its better performance in motility-related parameters, which are important for fertilization (Dementieva et al., 2024).

Interestingly, we observed that the expression of AQP7 was significantly higher in Y sperm than in X sperm, which is consistent with previous studies in dairy cattle and goats (Huanshan, 2022; Shufang, 2021). AQP7, a critical member of the aquaglyceroporin family, facilitates the transport of water and glycerol across the sperm plasma membrane. This function is essential for maintaining osmotic balance during the freeze-thaw process (Delgado-Bermúdez et al., 2019a). The high permeability of AQP7 to glycerol, a commonly used cryoprotectant, enables efficient intracellular equilibration (Delgado-Bermúdez et al., 2019b). This mitigates osmotic stress and reduces the formation of intracellular ice crystals, thereby minimizing membrane damage and preserving sperm integrity. Proteomic studies in cattle have demonstrated that AQP7 is differentially expressed in sperm populations with varying cryotolerance. High AQP7 expression correlates with improved post-thaw sperm quality, including motility, acrosomal integrity, and plasma membrane stability, underscoring its potential as a molecular marker for selecting sperm with superior cryotolerance (Prieto-Martínez et al., 2017). Similar findings have been reported in boar sperm, where AQP7 expression was associated with enhanced glycerol transport and reduced oxidative stress, highlighting its protective role against cryopreservation-induced damage (Prieto-Martinez et al., 2017). These insights suggest that AQP7 may also play a significant role in the cryotolerance of sexed sperm. X- and Y-bearing sperm differ in their physical and biochemical properties, which may influence their responses to cryopreservation. Evidence from studies on sexed semen indicates that Y-bearing sperm exhibit superior cryotolerance compared to X-bearing sperm, potentially due to higher AQP7 expression. This hypothesis aligns with observations in non-sexed sperm (Fujii et al., 2018), where elevated AQP7 expression was linked to enhanced survival and functionality post-thaw. Further research is warranted to validate the role of AQP7 in the cryotolerance of sexed sperm and elucidate its molecular mechanisms. These studies could provide valuable insights into optimizing cryopreservation protocols (Jahanseir et al., 2024) and improving the efficiency of sexed semen production, ultimately contributing to the advancement of livestock reproductive technologies.

In contrast, our study demonstrated that the expression and cellular localization of AQP3 did not differ significantly between X and Y sperm. This finding is consistent with previous studies in cattle (Prieto-Martínez et al., 2017). However, research on boar sperm has suggested that AQP3 could serve as a biomarker for cryotolerance (Prieto-Martinez et al., 2017), although the underlying molecular mechanisms remain unclear. AQP3, as a member of the aquaporin family, is known to facilitate water and glycerol transport, similar to AQP7. While AQP3 and AQP7 share functional similarities, our findings suggest that AQP3 may have a less critical role in cryotolerance compared to AQP7. This difference may be attributed to the specific roles of these proteins in sperm physiology, including their subcellular localization and permeability characteristics (O'Brien et al., 2022; Pequeño et al., 2023). The distinct functionality and localization patterns of AQP7 and AQP3 further underscore the critical role of AQP7 in enhancing the cryotolerance of Y sperm. While AQP7 is more prominently associated with osmotic regulation and glycerol transport during cryopreservation, the contribution of AQP3 may be limited or context-dependent across species. These observations highlight the need for additional studies to elucidate the precise mechanisms by which aquaporins influence sperm cryotolerance and to determine species-specific variations.

## 5 Conclusion

The study suggests that the superior cryotolerance of Y sperm may be linked to specific proteins, particularly AQP7, which plays a pivotal role in protecting sperm from freezing-induced damage. This study provides valuable insights into the molecular mechanisms behind the differences in cryotolerance between X and Y sperm and may serve as a foundation for optimizing sexed semen production. Future research should further explore the role of AQP7 and other related proteins in enhancing sperm quality and improving artificial insemination techniques. Moreover, understanding these molecular mechanisms could also contribute to improving fertility treatments and the preservation of genetic resources in livestock breeding.

## Data Availability

The raw data supporting the conclusions of this article will be made available by the authors, without undue reservation.

## References

[B1] Arega A. Chalchissa G. (2019). Calves sex ratio in artificial and naturally bred cattle at Adami Tulu agricultural research center. J. Biol. Agric. Healthc. 9, 1–5. 10.7176/JBAH/9-1-01

[B2] Argov N. Sklan D. Zeron Y. Roth Z. (2007). Association between seasonal changes in fatty-acid composition, expression of VLDL receptor and bovine sperm quality. Theriogenology 67 (4), 878–885. 10.1016/j.theriogenology.2006.10.018 17157373

[B3] Ashrafzadeh A. Yajit N. L. M. Nathan S. Othman I. Karsani S. A. (2024). Comprehensive study of sperm proteins and metabolites potentially associated with higher fertility of zebu cattle (*Bos indicus*) in tropical areas. J. Proteome Res. 24 (1), 368–380. 10.1021/acs.jproteome.4c00926 39591502

[B4] Berger P. J. Cubas A. C. Koehler K. J. Healey M. H. (1992). Factors affecting dystocia and early calf mortality in Angus cows and heifers. J. Animal Sci. 70 (6), 1775–1786. 10.2527/1992.7061775x 1634401

[B5] Berry D. P. Cromie A. R. (2007). Artificial insemination increases the probability of a male calf in dairy and beef cattle. Theriogenology 67 (2), 346–352. 10.1016/j.theriogenology.2006.08.003 16979752

[B6] Cesari A. Cacciato C. S. De Castro R. E. Sánchez J. J. (2004). Partial purification and characterization of a trypsin-like serine protease from bovine sperm. Int. J. Androl. 27 (5), 311–318. 10.1111/j.1365-2605.2004.00484.x 15379973

[B7] Coronel C. E. Maldonado C. Aoki A. Lardy H. A. (1995). Electron microscopic immunolocalization of caltrin proteins in Guinea pig seminal vesicles. Archives Androl. 35 (3), 233–246. 10.3109/01485019508987876 8585779

[B8] Crews D. H. (2006). Jr: age of dam and sex of calf adjustments and genetic parameters for gestation length in Charolais cattle. J. animal Sci. 84 (1), 25–31. 10.2527/2006.84125x 16361488

[B9] Delgado-Bermúdez A. Llavanera M. Fernández-Bastit L. Recuero S. Mateo-Otero Y. Bonet S. (2019b). Aquaglyceroporins but not orthodox aquaporins are involved in the cryotolerance of pig spermatozoa. J. animal Sci. Biotechnol. 10, 1–12. 10.1186/s40104-019-0388-8 PMC6791021 31636902

[B10] Delgado-Bermúdez A. Noto F. Bonilla-Correal S. Garcia-Bonavila E. Catalán J. Papas M. (2019a). Cryotolerance of stallion spermatozoa relies on aquaglyceroporins rather than orthodox aquaporins. Biology 8 (4), 85. 10.3390/biology8040085 31726707 PMC6955868

[B11] Dementieva N. V. Dysin A. P. Shcherbakov Y. S. Nikitkina E. V. Musidray A. A. Petrova A. V. (2024). Risk of sperm disorders and impaired fertility in frozen–thawed bull semen: a genome-wide association study. Animals 14 (2), 251. 10.3390/ani14020251 38254422 PMC10812825

[B12] Elango K. Karuthadurai T. Kumaresan A. Sinha M. K. Ebenezer Samuel King J. P. Nag P. (2023). High-throughput proteomic characterization of seminal plasma from bulls with contrasting semen quality. Biotech 13 (2), 60. 10.1007/s13205-023-03474-6 PMC9877259 36714547

[B13] Fujii T. Hirayama H. Fukuda S. Kageyama S. Naito A. Yoshino H. (2018). Expression and localization of aquaporins 3 and 7 in bull spermatozoa and their relevance to sperm motility after cryopreservation. J. Reproduction Dev. 64 (4), 327–335. 10.1262/jrd.2017-166 PMC6105742 29798965

[B14] Gacem S. Castello-Ruiz M. Hidalgo C. O. Tamargo C. Santolaria P. Soler C. (2023). Bull sperm SWATH-MS-based proteomics reveals link between high fertility and energy production, motility structures, and sperm–oocyte interaction. J. Proteome Res. 22 (11), 3607–3624. 10.1021/acs.jproteome.3c00461 37782577 PMC10629479

[B16] Hinsch K.-D. De Pinto V. Aires V. A. Schneider X. Messina A. Hinsch E. (2004). Voltage-dependent anion-selective channels VDAC2 and VDAC3 are abundant proteins in bovine outer dense fibers, a cytoskeletal component of the sperm flagellum. J. Biol. Chem. 279 (15), 15281–15288. 10.1074/jbc.M313433200 14739283

[B17] Huanshan H. (2022). The identification of differentially expressed proteins of X and Y sperm and the functional study on DPY30 in dairy goat. In: northwest A&F University.

[B18] Jahanseir F. Ghasemian F. Zahiri Z. (2024). Aquaporin expression in cryopreserved human sperm: exploring the capabilities of natural deep eutectic solvents (NADES). Cryoletters 45 (3), 158–167. 10.54680/fr24310110512 38709187

[B19] Kasimanickam V. Kasimanickam R. (2024). MicroRNAs and their associated genes regulating the acrosome reaction in sperm of high-versus low-fertility Holstein bulls. Animals 14 (6), 833. 10.3390/ani14060833 38539931 PMC10967381

[B20] Kodzik N. Ciereszko A. Szczepkowski M. Karol H. Judycka S. Malinowska A. (2023). Comprehensive proteomic characterization and functional annotation of siberian sturgeon seminal plasma proteins. Aquaculture 568, 739326. 10.1016/j.aquaculture.2023.739326

[B21] Lorente G. Ntostis P. Maitland N. Mengual L. Musquera M. Muneer A. (2021). Semen sampling as a simple, noninvasive surrogate for prostate health screening. Syst. Biol. Reproductive Med. 67 (5), 354–365. 10.1080/19396368.2021.1923086 34180329

[B22] Mendes A. J. Murphy M. R. Casper D. P. Erickson P. S. (2022). The female to male calf sex ratio is associated with the number of services to achieve a calf and parity of lactating dairy cows. Transl. Animal Sci. 6 (3), txac080–4. 10.1093/tas/txac080 PMC9249140 35795071

[B23] Moore S. G. Hasler J. F. (2017). A 100-Year Review: reproductive technologies in dairy science. J. Dairy Sci. 100 (12), 10314–10331. 10.3168/jds.2017-13138 29153167

[B24] Mostek A. Janta A. Ciereszko A. (2020). Proteomic comparison of non-sexed and sexed (X-bearing) cryopreserved bull semen. Animal Reproduction Sci. 221, 106552. 10.1016/j.anireprosci.2020.106552 32861114

[B25] O'Brien E. Malo C. Castaño C. García-Casado P. Toledano-Díaz A. Martínez-Madrid B. (2022). Sperm freezability is neither associated with the expression of aquaporin 3 nor sperm head dimensions in dromedary camel (*Camelus dromedarius*). Theriogenology 189, 230–236. 10.1016/j.theriogenology.2022.06.029 35797755

[B26] Pequeño B. Martínez-Madrid B. Castaño C. Toledano-Díaz A. Bóveda P. Esteso M. C. (2023). Location of aquaporins 3, 7 and 10 in frozen-thawed ejaculated and cauda epididymal spermatozoa from the Iberian ibex, mouflon, and chamois. Theriogenology Wild 2, 100025. 10.1016/j.therwi.2023.100025

[B27] Pinart E. Yeste M. Bonet S. (2015). Acrosin activity is a good predictor of boar sperm freezability. Theriogenology 83 (9), 1525–1533. 10.1016/j.theriogenology.2015.02.005 25748245

[B28] Pranomphon T. Mahé C. Brair V. L. Parnpai R. Mermillod P. Bauersachs S. (2024). Oviduct epithelial spheroids during *in vitro* culture of bovine embryos mitigate oxidative stress, improve blastocyst quality and change the embryonic transcriptome. Biol. Res. 57 (1), 73. 10.1186/s40659-024-00555-5 39438935 PMC11494963

[B29] Prieto-Martínez N. Morató R. Muiño R. Hidalgo C. O. Rodríguez-Gil J. E. Bonet S. (2017). Aquaglyceroporins 3 and 7 in bull spermatozoa: identification, localisation and their relationship with sperm cryotolerance. Reproduction, Fertil. Dev. 29 (6), 1249–1259. 10.1071/RD16077 27221122

[B30] Prieto-Martinez N. Vilagran I. Morato R. Rivera Del Alamo M. M. Rodriguez-Gil J. E. Bonet S. (2017). Relationship of aquaporins 3 (AQP3), 7 (AQP7), and 11 (AQP11) with boar sperm resilience to withstand freeze-thawing procedures. Andrology 5 (6), 1153–1164. 10.1111/andr.12410 28941027

[B31] Ramirez-Diaz J. Cenadelli S. Bornaghi V. Bongioni G. Montedoro S. M. Achilli A. (2023). Identification of genomic regions associated with total and progressive sperm motility in Italian Holstein bulls. J. Dairy Sci. 106 (1), 407–420. 10.3168/jds.2021-21700 36400619

[B32] Saadi H. A. S. van Riemsdijk E. Dance A. L. Rajamanickam G. D. Kastelic J. P. Thundathil J. C. (2013). Proteins associated with critical sperm functions and sperm head shape are differentially expressed in morphologically abnormal bovine sperm induced by scrotal insulation. J. proteomics 82, 64–80. 10.1016/j.jprot.2013.02.027 23500133

[B33] San Agustin J. T. Lardy H. A. (1990). Bovine seminal plasma constituents modulate the activity of caltrin, the calcium-transport regulating protein of bovine spermatozoa. J. Biol. Chem. 265 (12), 6860–6867. 10.1016/s0021-9258(19)39228-2 2324086

[B34] Shufang L. (2021). The membrane protein differences of X/Y spermatozoa in dairy cattle and dairy goats. Inner Mongolia University.

[B35] Suo J. Wang J. Zheng Y. Xiao F. Li R. Huang F. (2024). Recent advances in cryotolerance biomarkers for semen preservation in frozen form–A systematic review. Plos one 19, e0303567. 10.1371/journal.pone.0303567 38776323 PMC11111053

[B37] Zhang R. Chen Y. Bao P. Wu F. Liang C. Guo X. (2023). Proteomic analysis of high and low-motility frozen-thawed spermatozoa in yak provides important insights into the molecular mechanisms underlying sperm cryodamage. Theriogenology 211, 182–190. 10.1016/j.theriogenology.2023.08.016 37643503

[B38] Zhang R. Liang C. Guo X. Bao P. Pei J. Wu F. (2022). Quantitative phosphoproteomics analyses reveal the regulatory mechanisms related to frozen-thawed sperm capacitation and acrosome reaction in yak (Bos grunniens). Front. Physiology 13, 1013082. 10.3389/fphys.2022.1013082 PMC9583833 36277216

[B39] Zhang Z. Yang Y. Wu H. Zhang H. Zhang H. Mao J. (2017). Sodium-Hydrogen-Exchanger expression in human sperm and its relationship with semen parameters. J. Assisted Reproduction Genet. 34, 795–801. 10.1007/s10815-017-0898-2 PMC5445042 28432487

