# Supplementary material for: Comparative analysis of AQP7 expression and cryotolerance in X- and Y-chromosome bearing bovine sperm
Source: Front Cell Dev Biol. 2025 May 16;13:1582961. doi: 10.3389/fcell.2025.1582961 (PMC12123227; doi:10.3389/fcell.2025.1582961)
Supplement: Supplementary file 1 [file DataSheet1.pdf]

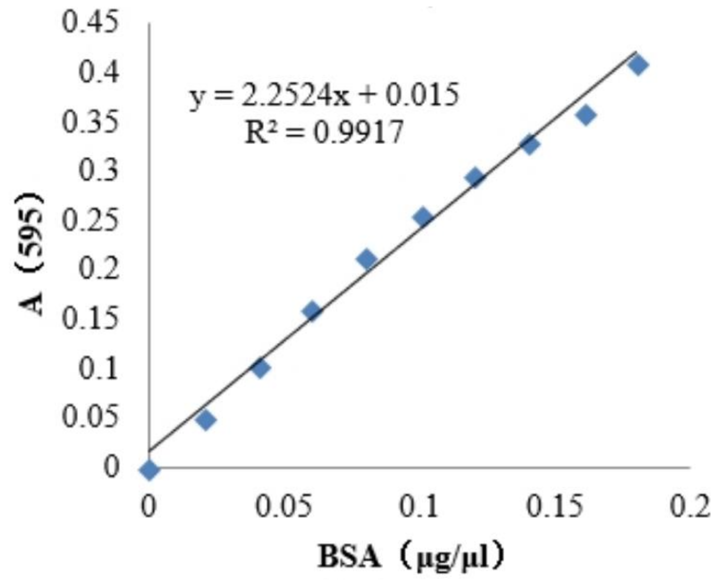

Figure S1: Protein Standard Curve of Bradford Protein Assay

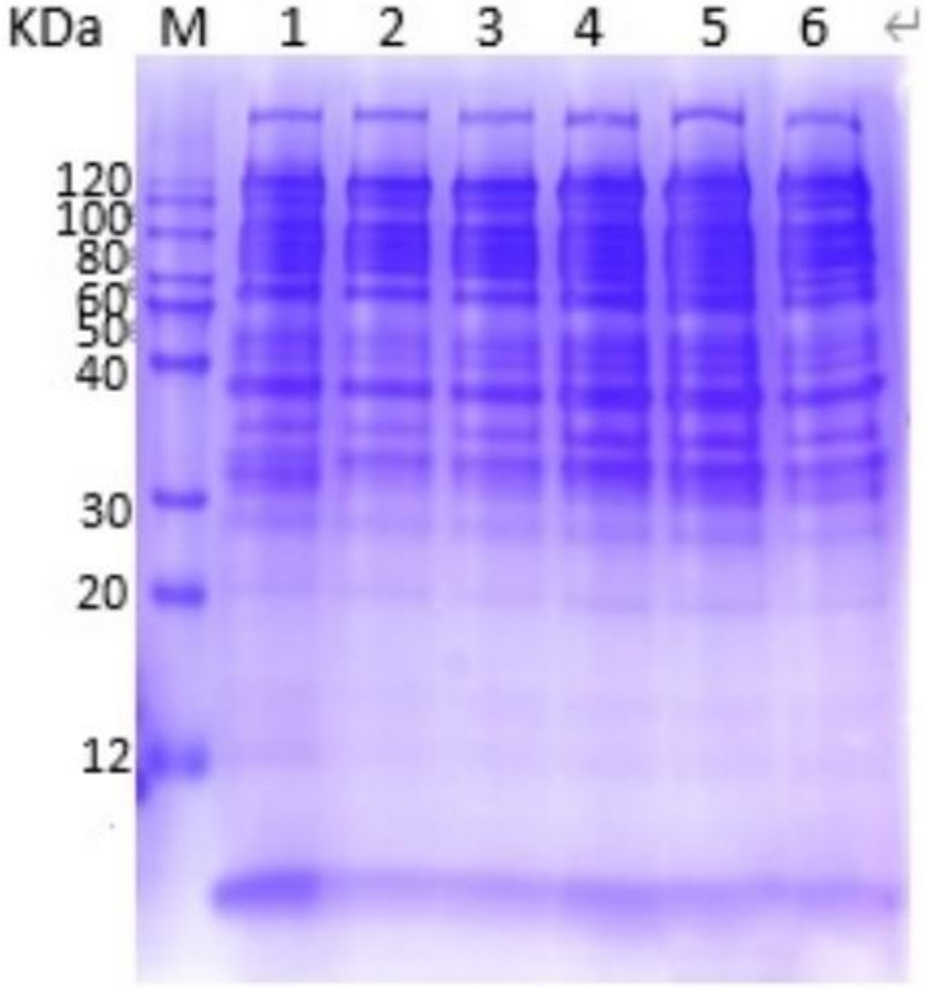

Figure S2. SDS-PAGE electrophoresis of X and Y sperm proteins

M: Marker; 1: AG 4600 X; 2: XM 9321 X; 3: HE 5104 X; 4: AG 4600 Y; 5: XM 9321 Y; 6: HE 5104 Y. Samples AG-4600-X, XM-9321-X, and HE-5104-X corresponded to X sperm, whereas AG-4600-Y, XM-9321-Y, and HE-5104-Y represented Y sperm

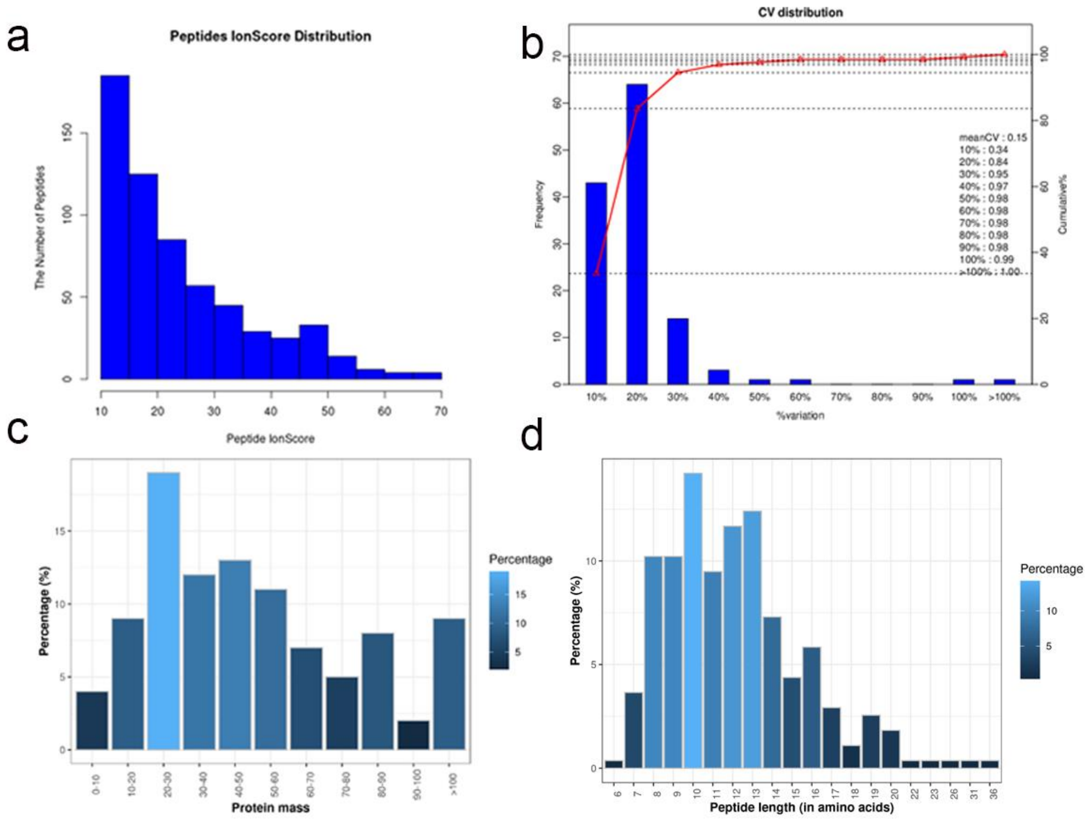

Figure S3. Mass spectrometry quality control

a) Peptide ion score distribution, b) CV distribution from repeated experiments, c) Protein molecular weight distribution, d) Peptide length distribution.

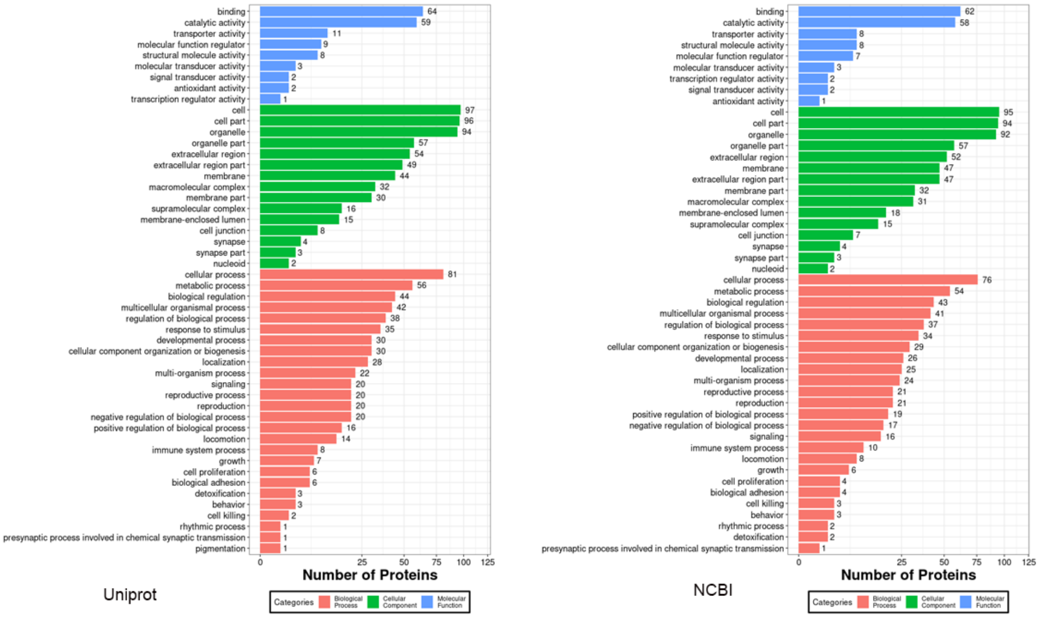

Figure S4. GO annotation chart for all identified sperm proteins

The bar graph shows GO annotations under three categories: molecular function, cellular component, and biological process. Different colors represent different GO categories.

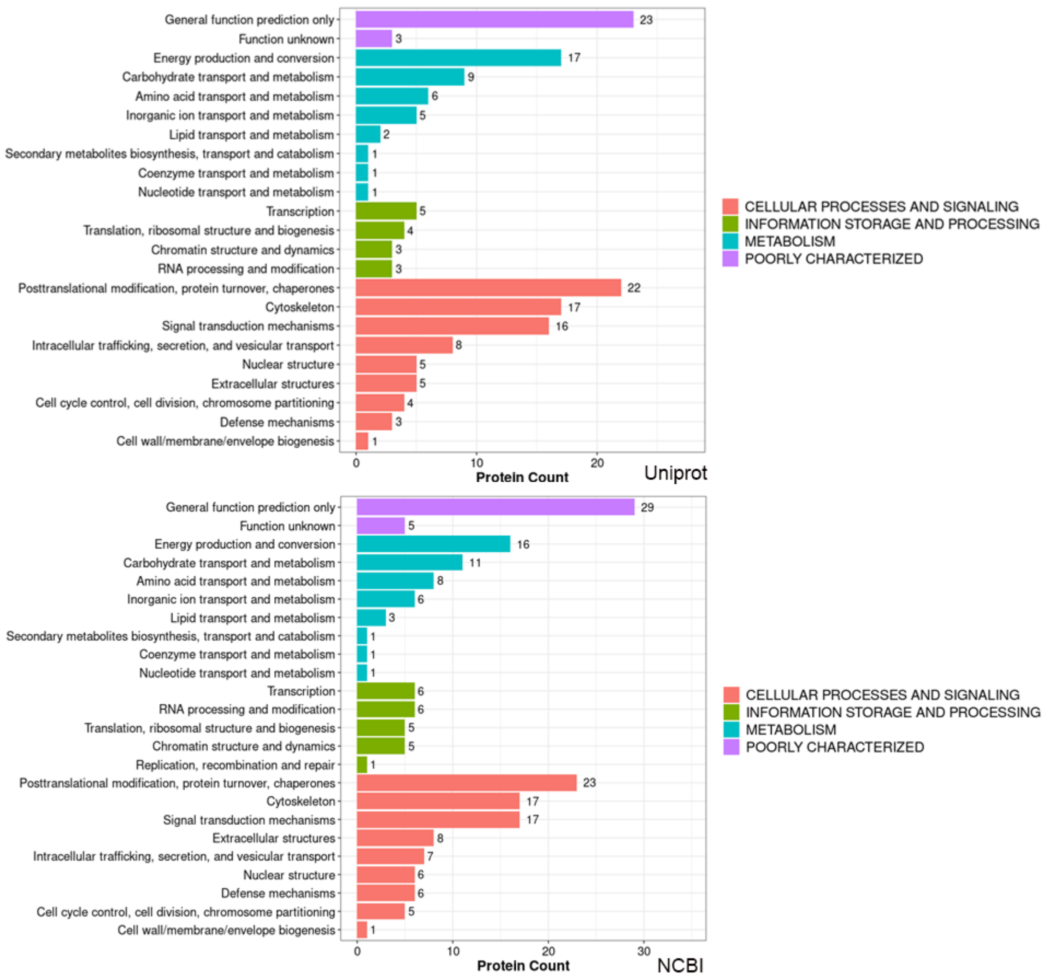

Figure S5. KOG annotation chart for all identified sperm proteins

X-axis: Number of proteins annotated to each KOG entry; Y-axis: Corresponding KOG categories.

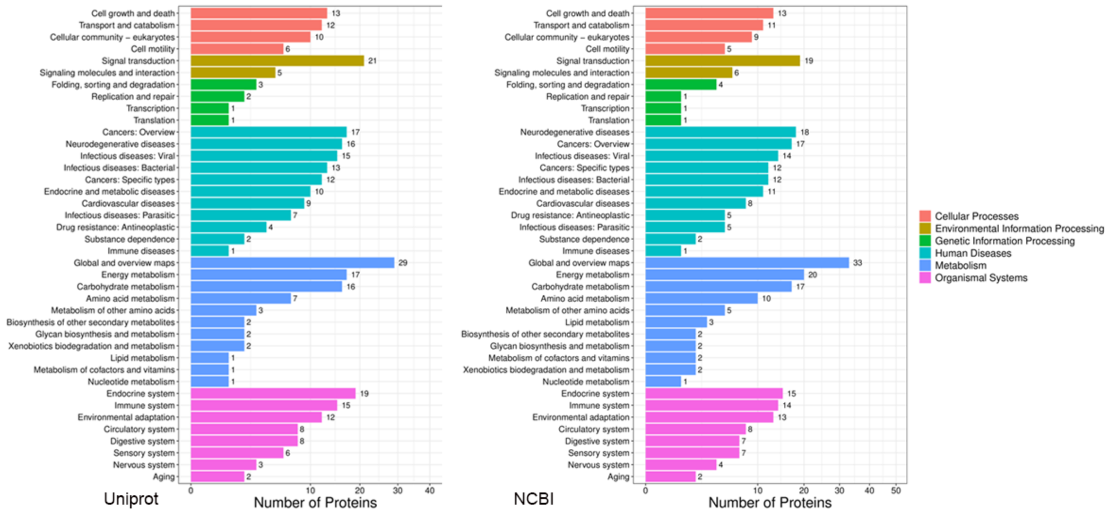

Figure S6. KEGG pathway annotation chart for all identified sperm proteins

X-axis: Number of proteins annotated in each KEGG category; Y-axis: KEGG function categories.

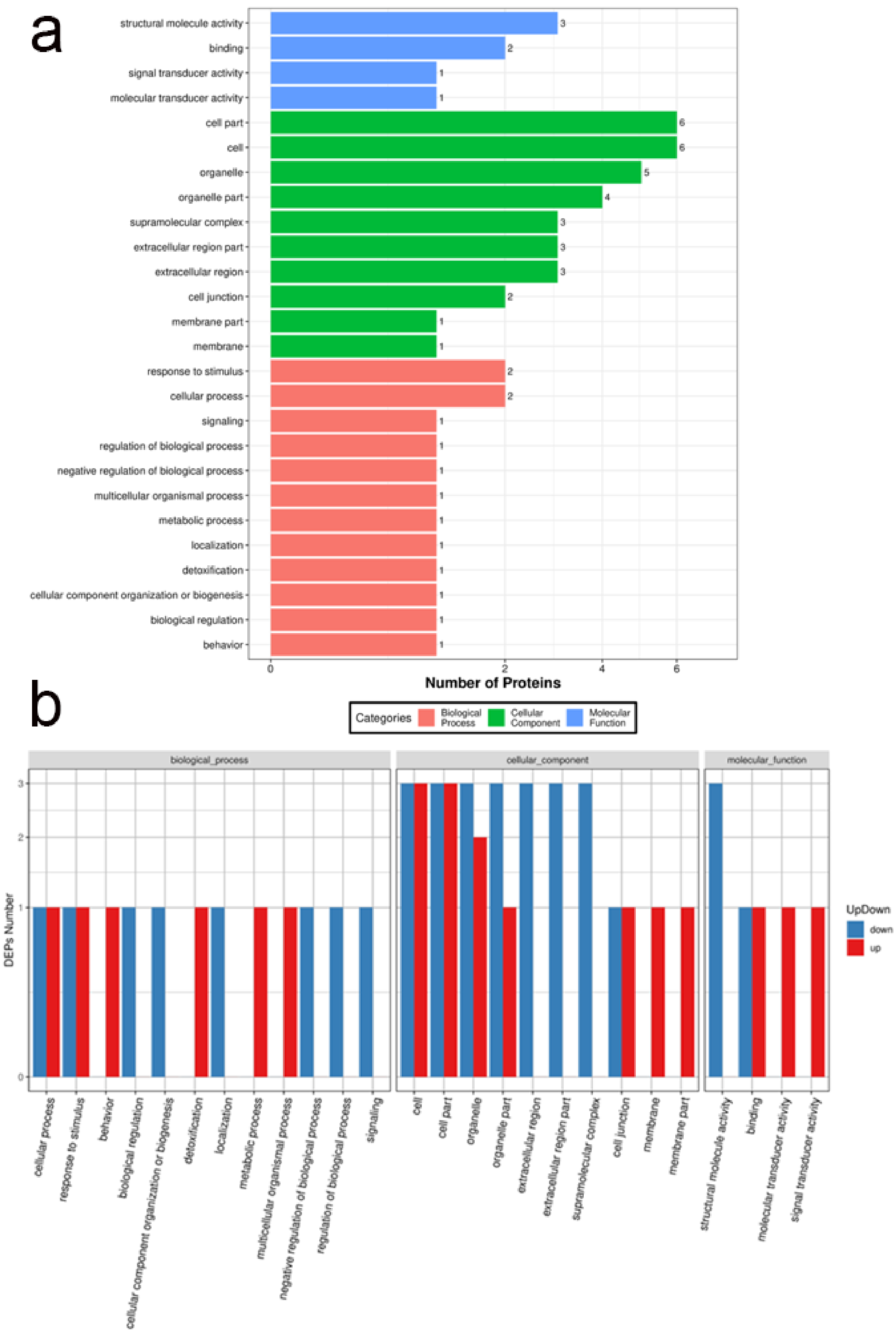

Figure S7. GO functional classification of differential proteins in X and Y sperm a) GO functional classification chart for differential proteins in XY sperm; b) Upregulation and downregulation statistics of GO functional categories.
